# Human immunodeficiency virus infection in pregnant women and its correlation with socioeconomic determinants

**DOI:** 10.1590/1980-220X-REEUSP-2022-0321en

**Published:** 2023-11-20

**Authors:** Lidiane de Nazaré Mota Trindade, Laura Maria Vidal Nogueira, Ivaneide Leal Ataíde Rodrigues, Ricardo José de Paula Souza e Guimarães, Maria Helena do Nascimento Souza

**Affiliations:** 1 Universidade Federal do Rio de Janeiro, Escola de Enfermagem Anna Nery, Rio de Janeiro, RJ, Brazil.; 2 Universidade do Estado do Pará, Escola de Enfermagem Magalhães Barata, Departamento de Enfermagem Comunitária, Belém, PA, Brazil.; 3 Instituto Evandro Chagas, Laboratório de Geoprocessamento, Ananindeua, PA, Brazil.; 4 Universidade Federal do Rio de Janeiro, Escola de Enfermagem Anna Nery, Departamento de Enfermagem e Saúde Pública, Rio de Janeiro, RJ, Brazil.

**Keywords:** HIV Infections, Pregnant Women, Spatial Analysis, Geographic Information Systems, Health Status Disparities, Infecciones por VIH, Mujeres Embarazadas, Análisis Espacial, Sistemas de Información Geográfica, Disparidades en el Estado de Salud, Infecções por HIV, Gestantes, Análise Espacial, Sistemas de Informação Geográfica, Desigualdades em Saúde

## Abstract

**Objective:**

To analyze the spatial pattern of human immunodeficiency virus infection in pregnant women and its correlation with socioeconomic determinants.

**Method:**

Ecological study, carried out with cases of human immunodeficiency virus infection in pregnant women in the state of Pará, Brazil, from 2010 to 2017. Rate analysis was performed using the empirical Bayesian method and univariate local Moran. Bivariate analyses were used to examine the correlation between infection and socioeconomic determinants.

**Results:**

High rates of infection were observed in municipalities in the mesoregions of Southeast of Pará and Metropolitan area of Belém. A significant spatial correlation was found between human immunodeficiency virus infection rates in pregnant women and human development index indicators (I = 0.2836; p < 0.05), average income (I = 0.6303; p < 0.05), and illiteracy rate (I = 0.4604; p < 0.05).

**Conclusion:**

The spatial pattern of human immunodeficiency virus infection in pregnant women correlated to socioeconomic determinants highlights the need to restructure public policies for the control and prevention of AIDS virus that take into account the socioeconomic factors of this specific population and locoregional disparities in Pará.

## RESUMO

**Objetivo:**

Analisar o padrão espacial da infecção pelo vírus da imunodeficiência humana em gestantes e sua correlação com os determinantes socioeconômicos.

**Método:**

Estudo ecológico, realizado com casos de infecção pelo vírus da imunodeficiência humana em gestantes no estado do Pará, Brasil, de 2010 a 2017. A análise das taxas foi realizada por meio do método bayesiano empírico e Moran local univariado. As análises bivariadas foram empregadas para examinar a correlação entre a infecção e os determinantes socioeconômicos.

**Resultados:**

Verificaram-se altas taxas da infecção em municípios das mesorregiões Sudeste Paraense e Metropolitana de Belém. Identificou-se correlação espacial significativa entre as taxas de infecção pelo vírus da imunodeficiência humana em gestantes e os indicadores índice de desenvolvimento humano (I = 0,2836; p < 0,05), renda média (I = 0,6303; p < 0,05) e taxa de analfabetismo (I = 0,4604; p < 0,05).

**Conclusão:**

O padrão espacial da infecção pelo vírus da imunodeficiência humana em gestantes correlacionada aos determinantes socioeconômicos reforça a necessidade de reestruturação de políticas públicas de controle e prevenção do vírus da AIDS que atentem para os fatores socioeconômicos desse público específico e disparidades locorregionais no Pará.

## RESUMEN

**Objetivo:**

Analizar el estándar espacial de la infección por el virus de la inmunodeficiencia humana en mujeres embarazadas y su correlación con determinantes socioeconómicos.

**Método:**

Estudio ecológico, realizado con casos de infección por el virus de la inmunodeficiencia humana en mujeres embarazadas en el estado de Pará, Brasil, de 2010 a 2017. El análisis de tasas se realizó mediante el método bayesiano empírico y Moran local univariado. Se emplearon análisis bivariados para examinar la correlación entre la infección y los determinantes socioeconómicos.

**Resultados:**

Se observaron altas tasas de infección en municipios de las mesorregiones Sudeste de Pará y Metropolitana de Belém. Se identificó una correlación espacial significativa entre las tasas de infección por el virus de la inmunodeficiencia humana en mujeres embarazadas y los indicadores del índice de desarrollo humano (I = 0,2836; p < 0,05), ingreso medio (I = 0,6303; p < 0,05) y tasa de analfabetismo (I = 0,4604; p < 0,05).

**Conclusión:**

El estándar espacial de la infección por el virus de la inmunodeficiencia humana en mujeres embarazadas correlacionado con determinantes socioeconómicos refuerza la necesidad de reestructurar políticas públicas para el control y la prevención del virus del SIDA que tengan en cuenta los factores socioeconómicos de esta población específica y las disparidades locorregionales en Pará.

## INTRODUCTION

The global response to infection by the Human Immunodeficiency Virus (HIV), the etiological agent of acquired immunodeficiency syndrome (AIDS), has been declining in recent years^(1)^, reinforcing the challenges of meeting the Sustainable Development Goals (SDGs), proposed by the United Nations (UN), which aim to end the AIDS epidemic by 2030^(2)^.

The increase in the number of HIV cases in pregnant women has received special attention in the planning of HIV/AIDS prevention and control actions, as vertical transmission of the virus is the main source of infection in children under 13 years of age^(3)^.

In Brazil, from 2000 to June 2021, 141,025 pregnant women infected with HIV were reported. Between 2010 and 2020, there was a 30.3% increase in the HIV detection rate in pregnant women in the country (rising from 2.1 cases per 1,000 live births to 2.7 cases per 1,000 live births). This increase can be explained, in part, by the expansion of prenatal diagnosis due to greater access to rapid tests, and the improvement of epidemiological surveillance of vertical HIV transmission^(4)^.

In the same period, the North region stood out on the national scene as the one with the highest increase in the rate of HIV in pregnant women, corresponding to an increase of 111.3%. Following the epidemiological panorama of infection in the region, the state of Pará presented, in 2020, the eighth highest rate of HIV detection in pregnant women among the 27 units of the Federation, recording a rate of 3.3 cases per 1,000 live births^(4)^.

Given the magnitude of this problem in Pará, more extensive investigation into the conditions and determinants of HIV in this specific population is required, to improve morbidity and mortality indicators from the disease in the State^(5)^.

Recent studies point to the significant role of the territory’s socioeconomic indicators as determinants of HIV infection, as social and economic conditions can interfere, positively or negatively, in the health of population groups^(5,6)^. However, research whose object addresses HIV in pregnant women, associated with socioeconomic determinants, is still little explored, especially in the North of Brazil^(6)^, a region that showed the highest growth in the rate of HIV detection in pregnant women in the last decade^(4)^.

Likewise, the use of geoprocessing tools and spatial analysis techniques in mapping HIV/AIDS cases in different territories has been successful in outlining priority areas for planning and programming disease prevention and control strategies, as well as in the evaluation of the actions carried out, resulting in a greater impact on risk conditions and morbidity indicators^(6-8)^.

Furthermore, it is understood that the correlation of the occurrence of HIV in pregnant women reported in the health information system with relevant spatial issues may identify spatial patterns that will support public managers in implementing targeted programmatic strategies and establishing new services, since mapping of infection, in this specific audience, can point out spatial clusters at high risk for vertical transmission, allowing more effective control and prevention of new cases of HIV^(9)^.

Likewise, a broader view of HIV infection in pregnant women, as well as the biological aspect, may contribute to reflection on the qualified care of health professionals, guiding them towards practices that are sensitive to the health needs of these women, favoring the development of critical competences for assisting pregnant women living with HIV/AIDS, which should not be taken out of context of the social reality in which they are inserted.

In view of the above, based on the hypothesis that the occurrence of cases of HIV infection in pregnant women is correlated with socioeconomic determinants, the objective of the study was to analyze the spatial pattern of human immunodeficiency virus infection in pregnant women and its correlation with socioeconomic determinants.

## METHOD

### Design of Study

This is an ecological, analytical study, guided by the methodological instrument *Strengthening the Reporting of Observational Studies in Epidemiology* (STROBE)^(10)^.

### Local

The study took place in Pará, Northern region of Brazil. The State is made up of 144 municipalities, distributed across six mesoregions: Baixo Amazonas, Marajó, Metropolitan region of Belém, Northeast Pará, Southeast Pará, Southwest Pará. A mesoregion is a regional geographic division, adopted by the Brazilian Institute of Geography and Statistics (IBGE), which brings together municipalities with similar geographic and socioeconomic characteristics. Their importance lies in the fact that they are regional spaces for integrated development actions that imprint a local identity^(11)^.

Pará is the second largest state in Brazil in terms of territorial extension, with 1,248,042.515 km^2^ and an estimated population of 8,777,124 inhabitants in 2021^(11)^.

### Population and Selection Criteria

The study population consisted of cases of pregnant women with HIV infection reported to the Notifiable Diseases Information System (*SINAN*), between 2010 and 2017, living in the state of Pará. Of the 2,923 notifications registered, 431 cases were excluded due to duplicity and/or incompleteness in the information field regarding the pregnant woman’s municipality of residence. Thus, 2,492 cases were eligible for the study.

### Data Collection

Data were collected in December 2018 after being requested from the State Coordination of Sexually Transmitted Infections/AIDS and Viral Hepatitis of the Public Health Secretary of Pará (*SESPA*), and were available in file format in the software Microsoft Office Excel^®^ 2010.

Data relating to socioeconomic indicators, such as the human development index (HDI), average income, illiteracy rate, and Gini coefficient, were obtained from the IBGE website through the Automatic Recovery Information System (*SIDRA*). The cartographic bases with municipal limits, mesoregions and states, in shapefile format (.shp), were also obtained from the aforementioned institute’s website.

### Data Analysis and Treatment

Initially, *SINAN*’s database was organized according to the variables of interest (notification number, age, education, occupation, and municipality of residence of the pregnant woman), then the information was decoded. For this, SINAN NET Version 5.0 data dictionary was used for HIV pregnant women.

To identify duplicates, all homonyms were checked, relating them to the pregnant women’s date of birth, mother’s name, and address for each case. Following confirmation of duplicity, the oldest notification was considered for analysis, with the filling of other fields being previously checked for information retrieval.

To characterize the population, the variables age, education, and occupation underwent descriptive analysis, the results of which were expressed in relative and absolute frequencies.

After this process, the geographic database (BDGeo) was built. The units of analysis were municipalities.

Calculations of HIV detection rates in pregnant women (per 1,000 live births) in the state of Pará and its municipalities were carried out by summing the cases during the study period, divided by the total number of live births in the same period^(5)^. To compose the denominators, we used the number of live births provided by the Live Birth Information System (*SINASC*), available on the DATASUS website.

To minimize the instability of gross rates, they were smoothed using the Local Empirical Bayesian method to correct random causal fluctuations that occur, especially, in small municipalities.

To calculate this estimate, the neighborhood matrix was used according to the contiguity criterion, in which the value “1” was assigned when the municipalities had common borders and “0” when they did not share borders^(6)^. The calculation of smoothed rates was performed in TerraView version 4.2.2 and the distribution map was produced in the software *ArcGis* ^®^ version 10.

Finally, to verify the correlation between the HIV detection rate in pregnant women and socioeconomic indicators, the Bivariate Local Moran index (I) was used. Thus, the hypothesis of “inverse” (I < 0) and “direct” (I > 0) spatial autocorrelation was assumed, and “randomness” (I = 0) was considered, with significance for p < 0.05. Strong spatial autocorrelation was considered for (I) close to –1 or 1.

This information was spatialized in the software GeoDa™ version 1.14, allowing the identification of 4 spatial cluster patterns (high-high, low-low, low-high, high-low). The high-high pattern refers to areas with high rates of HIV detection in pregnant women whose neighboring areas present relatively high socioeconomic indicators (MHDI, average income, illiteracy rate, and Gini coefficient). The low-low type pattern corresponds to areas with low HIV detection rates in pregnant women living in geographic areas with relatively low socioeconomic indices. Areas classified as low-high are those with low rates of HIV detection in pregnant women, whose neighborhoods have high socioeconomic indexes. Finally, high-low geographic areas are those with high rates of HIV detection in pregnant women living near areas with lower socioeconomic indicators.

### Ethical Aspects

This study was approved in 2018 by the Research Ethics Committee of the Undergraduate Nursing Course at the State University of Pará (UEPA), in accordance with the recommendations of Resolution 466/12 of the National Health Council, under opinion no. 2.997.808.

## RESULTS

A total of 2,492 cases of HIV in pregnant women in Pará were analyzed, whose HIV detection rate in this specific group was 2.2 cases/1,000 live births in the period. The cases had a mean age of 25 years (Standard deviation = ±6), the most prevalent age group was 20 to 29 years, with 59.9% (n = 1,498). Regarding education, it was found that 42.7% (n = 1,064) of pregnant women had less than 8 years of education. Regarding occupation, 46.5% (n = 1,159) of the cases were housewives.

**Figure 1:**
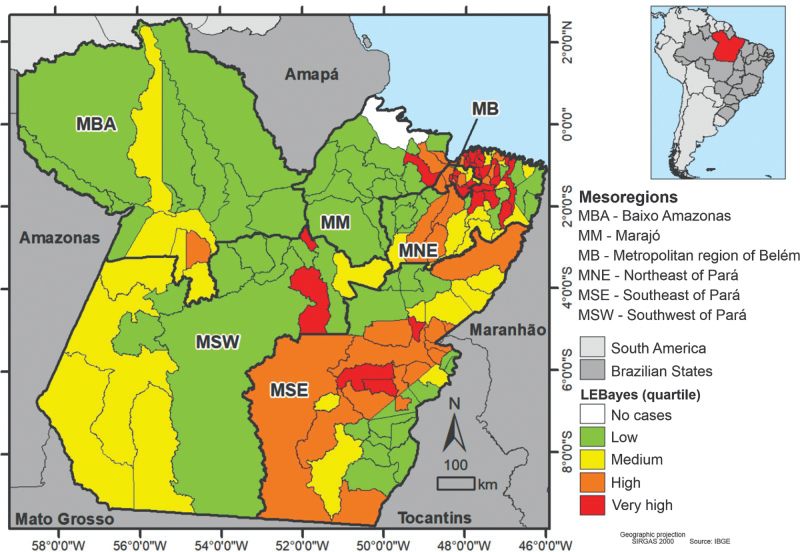
Spatial distribution of the HIV rate in pregnant women smoothed by the local empirical Bayesian method. Pará, Brazil, 2010–2017.

The spatial distribution of smoothed HIV detection rates in pregnant women/1,000 live births (Figure 1) highlighted municipalities with very high rates concentrated in the Southeast Pará, Northeast Pará, and Metropolitan region of Belém.

**Figure 2:**
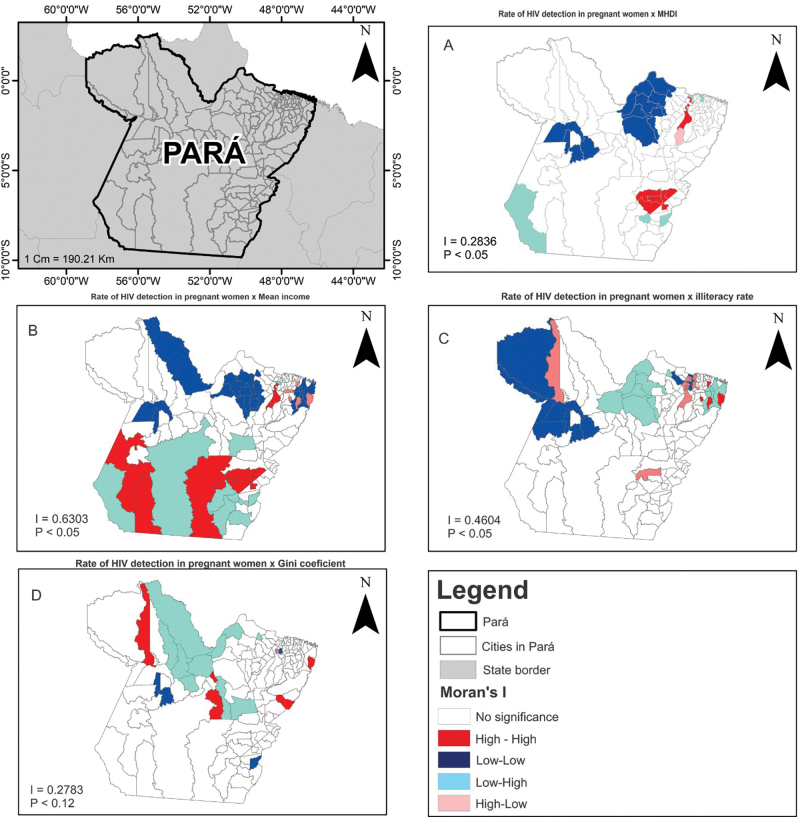
Spatial correlation between the HIV detection rate in pregnant women in the state of Pará and socioeconomic indicators. Pará, Brazil, 2010–2017.

The spatial correlation, obtained by the Moran index (I), between the HIV detection rate in pregnant women and the socioeconomic indicators HDI, average income, and illiteracy rate demonstrated significant spatial dependence (p-value < 0.05) among them, except with the Gini coefficient (I = 0.2783; p-value = 0.12), as seen in Figure 2.

The HIV detection rate among pregnant women, when ­associated with the HDI, showed a positive correlation index (I = 0.2836; p-value < 0.05), demonstrating a high-high spatial pattern concentrated in the municipalities of Southeastern Pará and the Metropolitan mesoregion of Belém, as shown in Figure 2A. Moreover, the formation of low-low pattern clusters in municipalities located in the mesoregion of Marajó was observed.

The HIV rate in pregnant women, associated with the ­average monthly income, showed a positive correlation index (I = 0.6303; p-value < 0.05), revealing a high-high clustering pattern in municipalities in the Southeast mesoregion of Pará. The low-low spatial pattern was observed in municipalities located, mainly, in the mesoregion of Marajó (Figure 2B).

The spatial autocorrelation of HIV rates in pregnant women and illiteracy rates was significant (I = 0.4604; p-value < 0.05), indicating the formation of a low-low cluster in municipalities located in the Baixo Amazonas mesoregion and Southwest mesoregion of Pará (Figure 2C).

## DISCUSSION

Pará has one of the highest HIV detection rates in pregnant women in Brazil^(4)^, showing the need for more efficient strategies to combat HIV/AIDS, to achieve the State’s programmatic goals and contribute to the fulfillment of the Sustainable Development Goals, referring to health and well-being, with regard to the eradication of AIDS and other epidemics by 2030^(2)^.

The spatial distribution of HIV rates in pregnant women shows higher rates in the Southeast and Metropolitan region of Belém mesoregions. The spatial pattern of HIV, in this population, showed a significant positive correlation with the socioeconomic determinants HDI, average income, and illiteracy rate. Thus, high HIV detection rates in pregnant women were seen in municipalities neighboring those with higher HDI and average income, while municipalities with lower HIV rates were surrounded by municipalities with low illiteracy rates.

The Southeast mesoregion of Pará has been marked by the intense process of urbanization, promoted by the expansion of mining industries, livestock farming activities and the construction of large projects, such as the Tucuruí hydroelectric plant, responsible for boosting the mobility of the population in search for job opportunities and better living conditions^(12,13)^. Likewise, the Metropolitan mesoregion of Belém offers a significant range of services and jobs, especially in the tertiary sector of the economy^(14)^.

The high rates of HIV in pregnant women, evidenced in the municipalities of the Southeast and Metropolitan region of Belém mesoregions, indicate the need to expand actions in these territories to prevent vertical transmission of the virus and reinforce the importance of serological screening carried out in prenatal care to detect the HIV and timely initiation of ART to minimize the risks of vertical transmission, especially in geographic areas with higher detection coefficients^(15-17)^.

However, research has reported weaknesses in the care provided during prenatal care, showing low testing coverage for HIV and other Sexually Transmitted Infections (STIs), even when pregnant women have six or more consultations^(9,18)^.

The high rates of HIV in pregnant women in municipalities with better HDI and average income identified in this study corroborate results from other research that demonstrated spatial dependence between HIV/AIDS epidemic hotspots and regions with high economic growth and better human development indices^(7,12)^.

These findings can be explained by the fact that municipalities with higher HDI have greater availability of resources and access to health services, as well as the implementation of new health care services, especially primary care. This increase in service coverage may have promoted greater access to rapid tests, timely diagnosis, and consequently, greater possibilities of case detection^(19)^.

It was also observed that municipalities in the Metropolitan area of Belém and Southeast of Pará mesoregions with higher HIV detection rates in pregnant women were located close to major highways, the construction of which is associated with the process of urbanization and economic development of these geographic spaces^(7)^.

The relationship between the increase in HIV cases and the presence of highways has been explained by the fact that they favor different changes in the social environment and play preponderant roles in the dissemination and maintenance of health problems and diseases in the surrounding population, since they act as links between different locations, making them conducive environments for greater social and sexual interaction^(7,20)^.

The results also demonstrated that municipalities with a low HDI, located in the Marajó mesoregion, have lower HIV detection rates in pregnant women. However, the possibility of underreporting of cases in these municipalities cannot be ruled out, given that the mesoregion has a low concentration of primary care services and one of the lowest prenatal care coverage in the state of Pará^(19,21)^. These aspects suggest the need to improve the actions of surveillance services in municipalities in this mesoregion, to increase the possibility of detecting new cases of HIV and producing epidemiological indicators that express the real health situation of the local population^(21)^.

The positive correlation between the HIV rate in pregnant women and average income can be explained by greater access to diagnostic exams and serological testing, peculiar in territories with better economic conditions^(22)^. However, a study carried out in the city of Recife showed that unfavorable economic conditions were statistically decisive for the increase in the HIV rate in pregnant women in that city^(23)^. This divergence in results confirms the heterogeneity of Brazilian regions and states in the epidemiological behavior of HIV/AIDS and the influence of socioeconomic determinants on population health^(6)^.

The results demonstrated the formation of clusters among municipalities with lower rates of HIV in pregnant women and low rates of illiteracy. It is accepted that low levels of formal education are directly related to the increase in HIV cases in low- and middle-income countries, such as Brazil^(15,24,25)^. Nevertheless, this relationship was not observed in this study, which found clusters formed by municipalities with high rates of HIV in pregnant women located in geographic regions close to areas with better levels of education.

A possible explanation for the disparity between the scientific literature and the results found in this study was the use of municipalities as a unit of analysis, since they can present, internally, very heterogeneous areas^(6,23)^.

The limitation of this study arises from the use of secondary data, which can be influenced by factors such as underreporting and incomplete information on *SINAN*’s notification forms, which can produce biases in the results. Anyway, the findings are robust, providing important contributions, especially to managers for the implementation of targeted programmatic strategies, taking into account the areas at greatest risk for vertical transmission. It also provides opportunities for reflection on the practice of health professionals regarding the qualification of prenatal care to minimize damage to maternal and child health.

## CONCLUSION

The results allowed observing that the pattern of HIV infection in pregnant women does not present a random distribution in the state of Pará and that the HIV detection coefficients in this specific female population have a positive spatial correlation with the socioeconomic determinants HDI, average income, and illiteracy rate. These findings point to the need to restructure public policies to prevent vertical transmission of the virus, valuing socioeconomic conditions and locoregional disparities in Pará.

It is expected that these results can support managers in adopting intersectoral political actions based on scientific evidence, as well as in identifying existing inequities in territories, favoring the evaluation of local surveillance programs and optimization of health professionals’ work processes, to obtain greater impacts on HIV/AIDS morbidity and mortality indicators.

The results also contribute to the reflection of health professionals on the importance of quality prenatal care that allows early diagnosis of HIV and treatment of pregnant women in a timely manner to control the epidemic and reduce mother-to-child transmission rates. It is also necessary for professionals who provide care to pregnant women with HIV to be aware of the influence of the territory and social context on these women’s health-disease process.

Finally, given the presence of possible social inequalities within the same municipality, it is recommended that new research be carried out that considers the analysis of smaller territorial units, such as neighborhoods and census sectors, to better understand the dynamics of HIV in pregnant women.
